# Blood Meal Analysis of *Culicoides* (Diptera: Ceratopogonidae) in Central Tunisia

**DOI:** 10.1371/journal.pone.0120528

**Published:** 2015-03-20

**Authors:** Darine Slama, Najoua Haouas, Habib Mezhoud, Hamouda Babba, Emna Chaker

**Affiliations:** 1 Department of Clinical Biology B, Laboratory of Parasitology-Mycology Medical and Molecular, University of Monastir, Monastir, Tunisia; 2 Laboratory of the Maternity and Neonatology centre of Monastir, Monastir, Tunisia; Institut Pasteur, FRANCE

## Abstract

To evaluate the host preferences of *Culicoides *species (Diptera: Ceratopogonidae) in Central Tunisia, we identified the source of blood meals of field collected specimens by sequencing of the cytochrome *b* (cyt *b*) mitochondrial locus and Prepronociceptine single copy nuclear gene. The study includes the most common and abundant livestock associated species of biting midges in Tunisia: *C*. *imicola*, *C*. *jumineri*, *C*. *newsteadi*, *C*. *paolae*, *C*. *cataneii*, *C*. *circumscriptus*, *C*. *kingi*, *C*. *pseudojumineri*, *C*. *submaritimus*, *C*. *langeroni*, *C*. *jumineri var *and some unidentified *C*. species. Analysis of cyt *b* PCR products from 182 field collected blood-engorged females’ midges revealed that 92% of them fed solely on mammalian species, 1.6% on birds, 2.4% on insects and 0.8% on reptiles. The blast results identified the blood origin of biting midges to the species level with exact or nearly exact matches (≥98%). The results confirm the presence of several *Culicoides* species, including proven vectors in Central Tunisia. Blood meal analyses show that these species will indeed feed on bigger mammals, thereby highlighting the risk that these viruses will be able to spread in Tunisia.

## Introduction

Abundance, longevity, frequency of feeding and host preference are key factors in determining the vector capacity of *Culicoides* species (Diptera: Ceratopogonidae) [1]. Host preference depends on the effort expended by the vector in the detection of blood meals and on the relative availability of suitable hosts [2]. Understanding the host preferences of *Culicoides* species can contribute to vector control and disease prevention. Host selection is influenced by environmental factors such as host availability, host diversity and distribution in the insect environment. Although many host preferences studies have been conducted on various mosquito and tick vectors [3, 4, 5], this has been neglected for biting midges until the outbreak and apparent overwintering of bluetongue (BT) [6] and Schmallenberg disease in Northern Europe [7].

*Culicoides* midges are small biting flies responsible for the transmission of several arboviruses global significance arboviruses as well as other pathogens of livestock [8, 9]. In the Mediterranean basin as well as sub-Saharan Africa, the main vector of bluetongue virus (BTV) and African horse sickness virus (AHSV) viruses that cause devastating diseases in ovine and equidae respectively, is *Culicoides imicola* [10]. Nevertheless, other Palaearctic *Culicoides* species, mainly within the subgenera *Avaritia* and *Culicoides*, such as *C*. *obsoletus*, *C*. *scoticus*, *C*. *dewulfi* and *C*. *pulicaris* are either known or potential BTV vectors [11, 12, 13, 14, 15, 16, 17].

The first outbreaks of BTV in Tunisia occurred in 1999 and two serotypes: BTV-2 and BTV-1 have arisen in 2000 and 2006, respectively [18]. Despite the fact that these outbreaks lead to a dramatic sanitary and economical crisis in Tunisia [19].

The host range of Mediterranean biting midges remains largely undetermined. Recent studies [20, 21, 22, 23, 24, 25], however indicate that these species acquire blood from a diverse range of mammals and birds, depending upon the relative number and availability of vertebrate hosts. Most *Culicoides* species are either mammalophilic or ornithophilic, although some feed on reptiles and frogs [8]. The longevity of adult biting midges is temperature dependent and has not been determined precisely for most species; it is suggested to last from a few weeks up to several months where recurrent blood meals occur [26]. This dependence on blood creates the situation where biting midges can transmit various pathogens. Thus, knowledge of the preference of *Culicoides* biting midges is crucial in assessing their vectorial capacity.

Recently, sensitive molecular-based assays have replaced earlier approaches based on antigen-antibody assays like precipitation test, latex agglutination and ELISA been developed to detect and identify blood meal sources of some insect vectors with a higher degree of accuracy [3]. Mitochondrial DNA such as cytochrome *b* or cytochrome oxidase I (*COI*) is referred because they exhibit a high level of interspecific polymorphism and for which a large set of data is available [26].

The purpose of the present study was to: i) identify the *Culicoides* species existent in Central Tunisia and ii) to assess the blood-feed behaviour of these biting midges by PCR detection and sequencing of the cytochrome *b* mitochondrial locus and Prepronociceptin (*PNOC*) single copy nuclear gene from field collected blood engorged females.

## Materials and Methods

### Ethics statement

The traps were placed on private property. All landowners were contacted before the field experiment, and all traps were set up with the permission from the landowners to conduct the study on their properties. The fieldwork did not involve any endangered or protected species. Materials used in the experiment posed no health risk to researchers or farmers and no vertebrate animals were harmed.

### Collection sites

**Fig 1:**
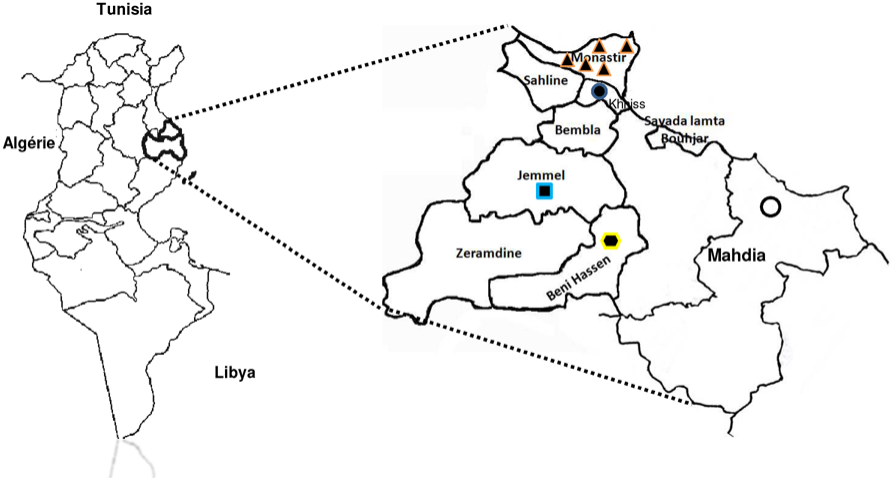
Localization of farms in Central Tunisia. Farm A is located in Khniss and reprented by a black circle with blue border. Farm B, C, D are located in Bir zira and showed by a black triangle with orange border. Farm E and F are identified by a black triangle with orange border also. Farm G is located in Jemmel and signified by a black square with a blue border. Farm H is showed by a lozenge with a yellow border. Farm N is situated in Mahdia and showed by a white circle with black border.

In order to determine the *Culicoides* species presence and abundance in the Central of Tunisia light trap collection were made on nine farms (Table 1, Fig. 1). The majority of sites were located on the governorat of Monastir and only one site was located on the governorat of Mahdia. The area of this region is 1 024 km2, with a human population of 542 100 (as 2013). The terrain is generally flat with many olive plantations. The coastal climate is semi arid, with annual average winter rainfall of 300 mm. The temperature is high in summer (avg = 40°C) and mild in winter. The temperature never falls below 0°C in winter and frost is absent. The animal fauna consists primarily of pet animals (dogs and cats) and livestock like cattle, sheep, horses and poultry. Two models of light traps: home-made miniature CDC (Centre of Disease Control, Atlanta, USA) and OVI (Onderstepoort Veterinary Institute) were used. All collections were done in human-inhabited biotopes where domestic animals (i.e., cattle, horses, dogs, goats, and chicken) are present (Table 1). The traps were installed no more than 1 m from the ground near to animals, either outside or inside shelters (Table 1). Traps were set before sunset and collected the following morning.

**Table 1:** Geographical and ecological characteristics of farms where blood-fed *Culicoides* were collected in Central Tunisia.

Farm code	Village	Grid reference	Collection date	Predominant animal species in the vicinity of the trap	Trap localisations
**Farm A**	Khniss	35°43'34", 10°49'34"	16/06/2009	cattle	Outside
**Farm B**	Bir zira	35°44'22", 10°48'49"	17/06/2009	cattle, sheep, chicken	Inside animal shelter
**Farm C**	Bir Zira	35°44'41", 10°49'77"	05/10/2009	sheep, chicken, turkey, dog	Outside
**Farm D**	Bir zira	35°46'15", 10°47'34"	12/10/2009	Cattle	Inside animal shelter, closed building
**Farm E**	Skanes	35°46'15", 10°47'34"	14/10/2009	sheep, chicken, turkey, goats	Outside
**Farm F**	Chaaba	35°45'51", 10°47'32"	19/10/2009	sheep, goats, dog	Outside
**Farm G**	Touza-jemmel	35°37'61", 10°49'65"	14/07/2010	sheep, cattle, goats	Outside
**Farm H**	Béni hassen	35°34'11", 10°48'87"	14/07/2010	sheep, chicken	Inside animal shelter
**Farm N**	Mahdia	35°30'76", 11°1'99"	12/06/2011	sheep, cattle, dog,	Inside animal shelter

### Midges sampling and identification

All insects were collected in a beaker filled with 70% ethanol. Sampled insects were transported to the laboratory. *Culicoides* midges were separated from other insects and identified according to wing characters using a stereo microscope. *Culicoides* specimens were subsequently subdivided into males, un engorged and engorged females (Table 2). Female blood-fed *Culicoides* were transferred in 90% ethanol.

**Table 2:** Total number of biting midges captured with light traps in Central Tunisia.

	Males	Females			Total
Farm A		Nulliparous	Parous	Engorged	
*C*. *jumineri*	0	4	0	8	12
*C*.*paolae*	1	0	0	0	1
*C*.*circumscriptus*	1	0	0	0	1
*C*.*newsteadi*	0	5	0	0	5
Farm B					
*C*. *imicola*	4	29	0	16	49
*C*. *jumineri*	0	0	0	26	26
*C*. *cataneii*	0	1	0	3	4
*C*. *circumscriptus*	0	11	0	2	13
*C*. *newsteadi*	0	1	0	0	1
*C*. *pseudojumineri*	0	1	0	0	1
Farm C					
*C*. *imicola*	90	223	557	123	993
*C*. *jumineri*	25	56	67	8	156
*C*. *newsteadi*	3	4	1	2	10
*C*. *paolae*	6	8	0	1	15
*C*. *submaritimus*	0	2	0	0	2
*C*. *jumineri var*	3	1	0	0	4
*C*. *circumscriptus*	0	1	0	0	1
*C*. *pseudojumineri*	3	6	0	0	9
*C*. *kingi*	0	3	0	0	3
Farm D, E					
*C*. *imicola*	0	5	0	1	6
*C*. *paolae*	2	1	0	1	4
Farm F					
*C*. *imicola*	400	1849	197	25	2471
*C*. *jumineri*	0	42	2	1	45
*C*. *langeroni*	0	3	0	0	3
*C*. *kingi*	0	0	2	0	2
*C*. *pseudojumineri*	0	8	0	0	8
*C*. *paolae*	0	6	0	0	6
*C*. *newsteadi*	0	2	0	0	2
Farm G					
*C*. *imicola*	1	0	0	0	1
*C*. *paolae*	679	64	12	9	764
*C*. *newsteadi*	5	60	0	0	65
*C*. *circumscriptus*	1	1	0	0	2
*C*. *jumineri*	0	1	0	0	1
Farm H					
*C*. *sp near kibunensis*	0	23	13	4	40
*C*. *kingi*	1	0	0	0	1
Farm N					
*C*. *jumineri*	6	60	6	5	77
*C*. *newsteadi*	0	4	1	2	7
*C*. *paolae*	2	0	2	0	4
*C*. *circumscriptus*	2	5	1	0	8
*C*. *cataneii*	0	1	0	0	1
Total	1235	2491	861	237	4824

Dissection was done with single-use sterile equipment. Head, wings and genitalia were removed in a drop of ethanol, mounted between slide and cover slide in a mix of Balsam Alcohol-phenol and incubated at 42°C for three weeks. Abdomens were transferred to individual sterilized 1,5mL vials and stored at -20°C before DNA extraction. Morphological identification was done according to their morphological characters [27, 28].

### Blood meal analysis

Only females with visible blood in the abdomen were assayed for the blood meal identification. Template DNA was extracted from individual engorged midges using the Qiamp DNA mini Kit (Qiagen, GmbH, Hilden, Germany) according to the manufacturer's instructions. The DNA was eluted in a final volume of 150 μL of AE buffer.

Sequences of primers used for the detection of host DNA are complementary to conserved regions of the vertebrate's cyt *b* gene [29]. In order to verify the first result a second marker was used. It was based on specific amplification and sequencing of the blood meal derived single copy prepronociceptin (*PNOC*) gene. This target is used in mammalian phylogenetic studies, and sequences of > 64 mammalian species are available on GenBank [30, 31].

Polymerase chain reactions (PCR) for cytochrome *b* and *PNOC* genes were performed in a 50 μL volume using 8 μL of the sample's extracted or control DNA; 2 units of Taq polymerase (Go Taq, Madison, WI), 10 μL of associated 2X buffer containing MgCl_2,_ 200 μM of dNTP and 1 μM of each of the following primers: cyt*b*1, 5'-CCA TCC AAC ATC TCA GCA TGA TGA AA-3'; cyt*b*2, 5'-GCC CCT CAG AAT GAT ATT TGT CCT CA-3' for cytochrome *b* [29] and *PNOC*-F: 5’-GCA TCC TTG AGT GTG AAG AGA A-3’, *PNOC*-R: 5’-TGC TCA TAA ACT CAC TGA ACC-3’ for *PNOC* [31, 32].

Two negative controls, distilled H_2_O, or DNA of biting midges (which do not feed on blood), were run simultaneously to detect possible contaminations both the extraction and amplification steps.

Amplification conditions for cytochrome *b* were: after an initial denaturation at 95°C for 10 min, 40 amplification cycles were performed (94°C for 30 s, 52°C for 30 s, 72°C for 45 s) and a final elongation at 72°C for 5 min. For *PNOC*, the initial denaturation step at 96°C for 8 min was followed by 50 cycles of 96°C for 30 s, 54°C for 30 s, 72°C for 30 s and a final elongation at 72°C for 5 min. Amplicons were analysed by electrophoresis in 1.5% agarose gel stained with ethidium bromide. PCR products were purified and sequenced in both directions using the same primers used for PCR (Eurofins MWG Operon, Munich, Germany).

### Sequences analysis

Sequences were edited using the Chromas software version 2.33 (http://ww.technelysium.com.au/chromas.html) and identified by comparison with the nucleotide-nucleotide Basic Local Alignment Search Tool (BLAST) (GenBank DNA sequence database, National Centre for Biotechnology Information) (www.ncbi.nlm.nih.gov/blast/) to assign unknown cyt *b* and *PNOC* sequences to a vertebrate species. Host species assignment was considered completed when we found a match of 98% or more between our sequences and those in GenBank.

### Evaluation of technical sensitivity on blood samples

To determine the technical sensitivity of the amplification of both cytochrome *b* and *PNOC* loci, 10-fold decreasing dilutions of optical density-quantified human DNA were used to test detection threshold.

### Accessions numbers

Sequences identified in this study have been deposited in GenBank under the following accession numbers: KP337884 (*Bos Taurus*), KP337885 (*Homosapien*s), KP337886 (*Ovis aries*), KP337887 (*Meleagris gallopavo*), KP337888 (*Canis lupus familiaris*) and KP337889 (*Capra hircus*) for *PNOC* gene and KP337890 (*Homosapien*s), KP337891 (*Capra hircus*), KP337892 (*Lanius Meridionalis*), KP337893 (*Aedes* sp), KP337894 (*Ovis aries*), KP337895 (*Canis lupus familiaris*), KP337896 (*Bos Taurus*), KP337897 (*Gallus gallus*) for cyt *b* gene. In total four sequences have not been deposited in GenBank because these were less than 200 base pairs (*Carlia fusca*, *Mustela nivalis*, *Musmusculus*, *Drosophila melanogaster*).

## Results

### *Culicoides* species presence and abundance in Central Tunisia

Of the 4824 midges collected 3589 were females. While some species were easily identified e.g. *C*. *circumscriptus* under the stereomicroscope, others could only be identified after dissection and microscope examination. At least twelve species of *Culicoides* were collected and identified (Table 2).

Diversity of *Culicoides* varied depending on site and/or date of collection (Table 1 and 2), reflecting the environmental differences in Central Tunisia. *Culicoides imicola* was the most abundant at all study sites, especially at the farm F. Indeed, it represents 51.2% (n = 2471) (Table 2). This species was trapped during October on a farm where the trap was placed outside (Table 1). In fact, the farm F was the most productive, seven species of *Culicoides* were trapped (Table 2). *Culicoides paolae* and *C*. *jumineri* were the second and third most abundant species in the same area with a relative abundance of 16.5% and 6.6% respectively (Table 2). A further eight species (*C*. *newsteadi*, 1.9%; *C*. sp, 0.8%; *C*. *circumscriptus*, 0.5%; *C*. *pseudojumineri*, 0.4%; *C*. *kingi*, 0.1%; *C*. *cataneii*, 0.1%; *C*. *jumineri var*, 0.1%; *C*. *langeroni*, 0.1% and *C*. *submaritimus*, <0.1%) represented only 4% of the collected midges.

### Blood meal identification

Only 237 of the 3 589 females collected were blood engorged. From these collections, 182 blood fed females were tested for blood meal origin after excluding 55 specimens with raptured abdomens or external contamination from other damaged blood fed arthropods. The majority of blood feds were collected in the outside trap at Farm C.

Of the 182 blood fed females tested, 125 (68.7%) were positive for cyt *b* DNA amplification (*C*. *imicola*, n = 96, *C*. *jumineri* n = 17, *C*. *newsteadi* n = 6, *C*. *cataneii* n = 1, *C*. *circumscriptus* n = 3, Unidentified *C*. sp n = 2) (Table 3). Negative controls (double distilled water and male midges) output no PCR products implying that only host’s DNA were amplified in the detection step.

**Table 3:** Origin of blood meals from blood-fed *Culicoides* spp.

	*C*. *jumineri*	*C*. *circumscriptus*	*C*. *newsteadi*	*C*. *imicola*	*C*. *cataneii*	Unidentified *C*.*sp*	Total
Mammals							
*Homo sapiens*	6	1	1	89	-	-	97
*Bos taurus*	6	-	-	-	-	-	6
*Capra hircus*	-	-	1	3	-	-	4
*Ovis aries*	-	-	1	2	-	-	3
*Mustela nivalis*	1	-	-	-	-	-	1
*Canis lupus familiaris*	-	-	-	1	-	-	1
*Mus musculus*	-	-	-	-	1	-	1
Birds							
*Meleagris gallopavo*	-	-	1	-	-	-	1
*Lanius meridionalis*	-	-	-	1	-	-	1
*Gallus gallus*	1	-	1	-	-	-	2
Insects							
*Drosophila melanogaster*	1	-	-	-	-	-	1
*Aedes* sp	1	-	-	-	-	1	2
Reptiles							
*Carlia fusca*	1	-	-	-	-	-	1
Unidentified blood meals	-	2	1	-	-	1	-
Total	17	3	6	96	1	2	125

All positive PCR products amplifying the cytochrome *b* gene were sequenced. Edited sequences were compared with GenBank database and identified to species level. Among these sequences, four of them were not assigned to any host (*C*. *circumscriptus* n = 2; *C*. *newsteadi* n = 1; Unidentified *C*. sp n = 1). No superposed fluorograms suggesting mixed blood meals were detected. The majority of the identified blood meal samples were from humans (*Homo sapiens*, n = 97, 77.6%), followed by cattle (*Bos taurus*, n = 6, 4.8%), goat (*Capra hircus*, n = 4, 3,2%), sheep (*Ovis aries*, n = 3, 2.4%), birds ([*Gallus gallus*, n = 2, 1.6%]; [*Lanius meridionalis*, n = 1, 0.8%]; [*Meleagris gallopavo*, n = 1, 0.8%]); dog (*Canis familiaris*, n = 1, 0.8%), rodents ([*Mus musculus*, n = 1, 0.8%]; [*Mustela nivalis*, n = 1, 0.8%]) and reptiles (*Carlia fusca*, n = 1, 0.8%) (Table 3). Beside, one blood fed was from insects [*Aedes* sp, n = 2, 1.6%] and other feeding come from *Drosphila melanogaster*, n = 1, 0.8% (Table 3).

Comparison of host availability at each trapping site and blood meal origins, indicate that the blood meals were taken on the hosts present. In fact, all six individuals find to be positive for cattle blood were collected on farms where cattle were the animals closets to the traps (Farm A, B, D, G and N) (Table 1). Although, the presence of cattle, sheep, chicken, dog and lambs in the vicinity to traps and to human-inhabited biotopes, *C*. *imicola* was found positive for *Homo sapiens* (n = 89), sheep, goat, dog and birds and have not fed on cattle (Table 3).

Results were confirmed by *PNOC* gene PCR-sequencing. In fact, among the 182 tested blood fed biting midges, only 24 were positive for the detection of this marker. This low percentage of detectable blood meal DNA by *PNOC* gene amplification is related to the target copy number (*PNOC* is a single copy gene). All amplification products were sequenced and blood meals were identified to species level.

We identify seven livestock species as mammalian hosts for *Culicoides* species. The majority of the blood meals were from humans (n = 16, 66.7%), followed by cattle (*Bos taurus*, n = 2, 8.3%), sheep (*Ovies aries*, n = 2, 8.3%), goats (*Capra hircus*, n = 1, 4.2%) and dog (*Canis lupus*, n = 1, 4.2%).

The *PNOC* PCR sequencing results obtained for the 24 sample confirmed that obtained by cyt *b* PCR sequencing. For one sample fluorogram obtained after sequencing of *PNOC* PCR product have no matches with any GenBank database sequences. However, this same sample was identified as *Melleagris gallopavo*, by using cyt *b* PCR sequencing method.

### Evaluation of technical sensitivity on blood samples

**Fig 2:**
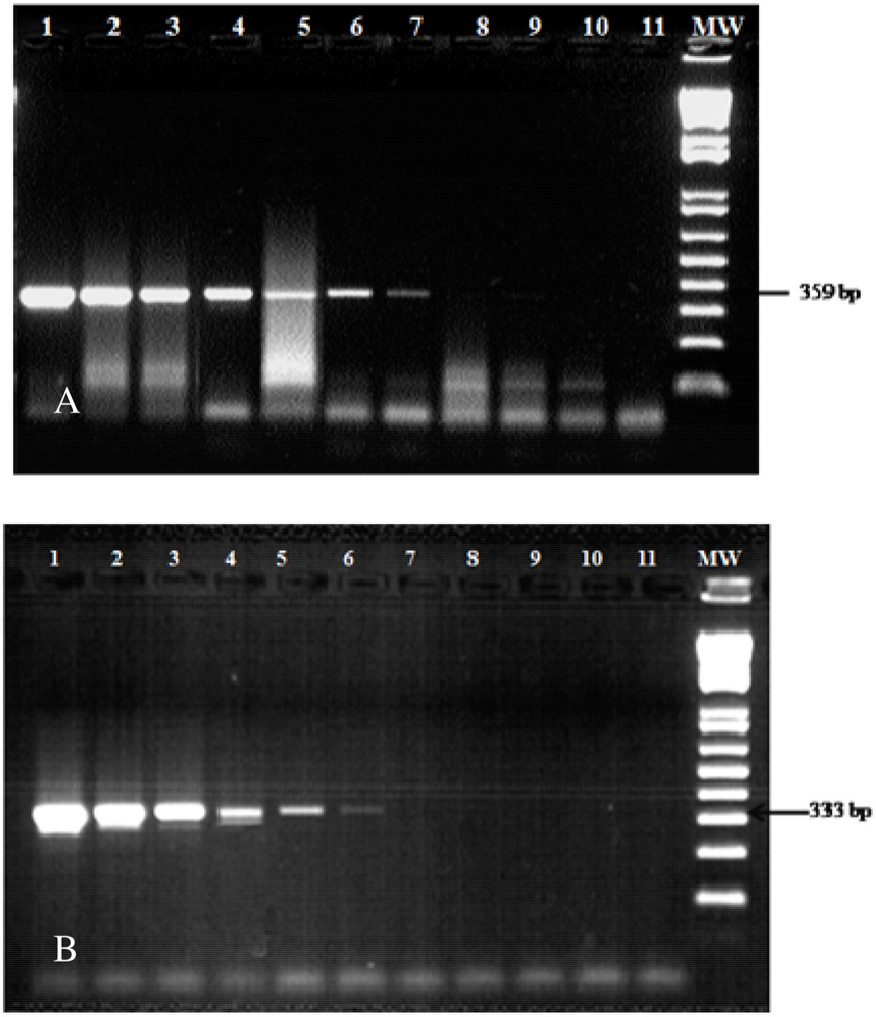
Analytical sensitivity of cyt *b* gene PCR amplification (359 bp) (A). Analytical sensitivity of *PNOC* gene PCR amplification (333 bp) (B). Lanes 1–10, 9-fold dilutions of human genomic DNA; Lane 11, negative control. MW, Molecular Weight marker (100-bp DNA ladder)

For cyt *b* gene PCR, a 359-bp PCR product was successfully detected from an estimated human genomic DNA quantity = 0.2 pg. However, for *PNOC* gene PCR, a 330 pb PCR product was detected from an estimated human genomic DNA quantity = 5.15 pg (Fig. 2. (A)-(B)).

## Discussion

Blood meal analysis revealed a specific host preference in biting midges for either mammals or birds, which are in general in agreement with published results [23, 33, 34, 35, 36]. *Culicoides* biting midges appear to feed opportunistically on their mammalian hosts, and their host selection generally seems to reflect host availability in the vicinity of the traps. We did not find evidence for host specificity beyond the separation of mammalophilic and ornithophilic species. However, the sample sizes of the ornithophilic species were small and, consequently, the possibility that some of these midges specialize on a restricted number of bird species cannot be ruled out [36].

Morphological identification revealed twelve species previously encountered in Central Tunisia. The most abundant blood-fed *Culicoides* species collected were *C*. *imicola*, *C*. *paolae* and *C*. *jumineri*. This finding agrees with those of previous surveys in Tunisia [37, 38], in which these species, were found to represent > 90% of all biting midges captured. It's noteworthy that *C*. *imicola*, the most abundant species in this study was absent at two of the nine farms sampled.

Human was the most prevalent host species sequenced, from the blood meals followed by cattle, goat, sheep, birds, dog, reptiles and rodents. This can most probably be ascribe the location of the traps in the direct vicinity of human habitats. In fact, we must highlight that the human soft skin with short or no hair could make this mammal species more attractive for feeding. Indeed as indicated in Table 1, all collections were done in the hot season when farmers spent their nights outside thereby giving the opportunity for *Culicoides* to bite them. Based on previous reports [39, 40, 41], the role of *Culicoides* midges on public health mostly occurs through nuisance biting inflicted by female, leading in severe cases to cutaneous pruritic wheal-and-flare responses and permanent scarring. Opportunistic feeding on humans by a wide range of *Culicoides* species has been documented; however, certain species have become notorious for this activity through their vast population density and persistent biting attacks, shaping public perception of the genus in many regions including northern Europe [42, 43, 44, 45]. With regard to disease transmission, the most important role of *Culicoides* midges in human health lies in their ability to biologically transmit Oropouche virus, the aetiological agent of the febrile illness Oropouche fever, between humans beings [41, 46, 47]. Oropouche virus is currently restricted to the Neotropics and infects humans, causing major outbreaks of febrile illness. The reservoir hosts are, however, poorly characterized [46].

The most abundant blood-fed midge species, *C*. *imicola*, have fed on humans, with only single specimens found to feed on goats and birds (Table 3). Previous studies seem to demonstrate opportunistic host preferences for *C*. *imicola* to be various bigger livestock species, although some individuals was found to have fed on birds [9]. We believe, therefore, that the classification of this biting midge species as mammalophilic opportunists is quite acceptable. Even so, the high percentage of *C*. *imicola* found to feed on humans in the current study is quite remarkable. The reason for this pheromone is no not clear and need to be investigate furhter. Opportunistic feeding behaviour in most mammalophilic *Culicoides* species has been suggested by Bartsch et al. [20] and Lassen et al. [22, 23].

Even more interesting is that a single *C*. *imicola* was found to feed on dogs. Despite that is a single case, this leads us to question the extent that dogs can act as hosts for *Culicoides* species. The current result corroborates the findings of Oura and Harrak [48] who suggested that *Culicoides* species could indeed infect dogs with BTV up to 21% of the population. Whilst Braverman and Chizov-Ginzburg [49] found that all 400 blood meals analysed from *Culicoides* species in Israel and Zimbabwe were negative for canine blood. In Siberia, severe midge attacks on dogs (*C*. *chiopterus*, *C*. *pulicaris* and *C*. *fascipennis*) are commonly reportedly [50]. Notwithstanding the controversy, it is clear that *Culicoides* species do not feed on dogs to the same extent that they feed on horses and other livestock, itremain possible that dogs could be an incidental host. It is known that dogs may die from AHSV after the ingestion of meat from animals that have died from AHSV. In South Africa, however, it has been reported that dogs were dying from AHSV without any evidence of ingestion of horsemeat [51]. This case has a significant practical implication for understanding the epidemiology of AHSV. Although vector-borne transmission is likely further investigations are required before conclusions can be drawn about possible vectors and epidemiology.

In this study, we highlight for the first time the preferences of *C*. *jumineri* in Central Tunisia. This species appear to be varied in their blood meal host and fed on cattle, sheep, humans, rodents and birds (Table 3). The fact that it feed on cattle and sheep render it an ideal vector for BTV.

Some studies, [52, 53] have proven that mosquitoes (*Aedes* sp.) in captivity are attracted to and feed on insect larvae to produce viable eggs. Moreover, other genera belonging to Ceratopogonidae are known to feed on the haemolymph from insects [23, 54, 55]. Thus, the hypothesis that *Culicoides* midges may react similarly when in need of protein cannot be discarded. In a recent study Ma et al. 2013 [56] produced a video of two anopheline mosquitoes that were attacked by *Culicoides anopheles* and demonstrated the act of a midge taking blood from an engorged mosquito.

In the present study we found that the midge (*C*. *jumineri*) has fed primarly on the mosquitoes and not on the blood inside them. In fact, we have not observed a red blood on the midgut of the midge which were supposed to have fed on the mosquitoes and fruit flies. Although Braverman et al. [57] have reported that *C*. *newsteadi* fed only on human and poultry at least, five host DNA species were detected in the present study (Table 3). This variation in blood feeding behaviour may be explained by two hypotheses. First, the decrease in one host availability or accessibility may favour individual biting midges that have an innate preference for alternative, more abundant, or accessible hosts. Second, the population may express an environmentally induced phenotypic plasticity, so that changes in host availability or accessibility modify host selection patterns without changes in innate host preferences [58].

Among the captured *Culicoides* species, two species were represented by less than three blood fed females with identified blood meal hosts (Table 3). It is therefore not possible to assess their host preference. This low sampling may be explained by the position of the traps relative to the ground. Blood-seeking flies may choose a particular level above ground and feed on a range of hosts encountered at that level [59, 60].

Previous studies have found that a large range of mammals species were the preferred host for biting midges such as cattle for *C*. *chiopterus*, *C*. *puntatus*, *C*. *obsoletus* [20, 21, 22] and sheep for *C*. *punctatus*, *C*. *obsoletus*, C. *festivipennis*, *C*. *pulicaris* and *C*. *newsteadi* [24]. However, in the current study, we highlighted an opportunistic feeding behaviour of *Culicoides* species which was directly related to the relative abundance of host species present in the area.

In the present study, the mean percentage of unamplified DNA host using cyt *b* PCR technique was 31. 3%. Nevertheless, Lassen et al. [22], Ninio et al. [21] and Garros et al. [24], have respectively reported 10%, 9% and 7.8% of non amplified DNA host. Many factors may explain this discrepancy, but the most likely are: **i)** the difference in the quantity of target DNA. In fact, the blood meal volume found in biting midges varies from 0.1 to 1μL [61] which directly influence the quantity of host DNA present in the sample. **ii)** The second factor that may affect the sensitivity of host DNA detection is the process of degradation of this last one.

In the present study we used two different molecular markers for blood meal identification (cyt *b* DNA sequencing and *PNOC* sequencing). Cytochrome *b* gene has been commonly used for the blood meal identification due to their high copy numbers and sufficient genetic variation at the primary sequence level among vertebrate taxa [62, 63, 64, 65, 66]. Also more than 8 000 cytochrome *b* gene sequences of vertebrate animals are available in the GenBank/EMB/DDBJ Database [65].

Regarding the *PNOC* PCR sequencing methods, the sensitivity of host DNA detection was lower than found by cyt *b* PCR method. This may be explained by two hypotheses: the first one is the class of tested target locus (single or multicopy locus). Actually, PCR detection sensitivity was proved to be higher when targeting multicopy loci like cyt *b* mitochondrial DNA (8 000 copy per cell) [65] than when using a single copy gene. The second one is the specificity of the used primers. In fact, the used primers for the amplification of the *PNOC* gene do not recognize birds and reptiles DNA [31]. Moreover, only *PNOC* genes of 65 mammalians species exist in Genbank Database.

As previously reported, no mixed blood meals are detected in any female tested in the present study [22, 21, 24]. Even so, the recognizing whether mixed feeding may occur with biting midges is crucial since feeding on more than one host species may facilitates mechanical transmission.

Our results show that despite the semi arid conditions several *Culicoides* species, including, proven vectors, are present in Central Tunisia. Blood meal analyses show that despite a plasticity in their host preferences most of these species will indeed feed on bigger mammals. There is therefore a risk of these viruses spreading in Tunisia.
